# Society Meetings

**Published:** 1913-05

**Authors:** 


### SOCIETY MEETINGS

---

Dr. W. Gilman Thompson of New York lectured on Industrial Diseases of New York State, with lantern demonstration, before the Buffalo Academy of Medicine on April 8, and before the Rochester Academy of Medicine on April 9. A dinner in his honor was given at the Buffalo Club and the Rochester Club, respectively, preceding the lecture. In Buffalo, representatives of the Tuberculosis Association and the Life Underwriters' Association of Buffalo were present. The dangers of the strenuous life of a lecturer were not mentioned by the distinguished guest. Dr. Thompson's paper will be published in a future number of this Journal.

---

The I. C. I. Chapter of the Nu Sigma Nu Fraternity held open house to its active and alumni members at its home, 246 Elmwood avenue, Buffalo, April 7.

---

The Elmira Academy of Medicine, at its meeting April 2, endorsed the reappointment of Dr. Eugene H. Porter as State Commissioner of Health, and memorialized Gov. Sulzer to that effect. The reappointment of a meritorious official, without regard to politics, is the best way to secure efficient service and to remove the odium of partisanship from our political system.

---

The Buffalo Association for the Relief and Control of Tuberculosis has recently elected the following officers: President, Irving S. Underhill; Vice-Presidents, E. J. Barcalo and Mrs. C. J. Hamlin; Treasurer, Harry T. Ramsdell; Directors, Hugh Kennedy, Thomas B. Lockwood, Frederic Almy, Dr. Burt J. Maycock, Dr. Francis E. Fronczak, Theodore M. Pomeroy, Proctor Carr, E. J. Barcalo, William E. Hill, Mrs. James W. Putnam, Mrs. Charles E. Baker, Hugo A. Brown, Miss Mary L. Morgan.

The report of John R. Shillady, executive secretary, shows that during the summer 165 patients were cared for at the open air camp. Fifteen children were cared for a day and night, with special nurse in charge.

---

The Alumni Association of the College of Medicine of Syracuse University will be held on the afternoon of June 11, the dinner being held in the evening. Commencement exercises of the entire university will be held June 12.

---

The Fourth International Congress of School Hygiene will be held in Buffalo, August 25-30. The officers are as follows: President, Charles W. Eliot, President Emeritus, Harvard University; Vice-Presidents, Dr. William H. Welch, Professor of Pathology, Johns Hopkins University; Dr. Henry P. Walcott, Chairman Massachusetts Board of Health; Honorary Vice-Presidents, Dr. Abraham Jacobi, Professor Emeritus, College of Physicians and Surgeons, Columbia University; William H. Burnham, Ph. D., Professor of Pedagogy and School Hygiene, Clark University; His Eminence, James Cardinal Gibbons, Archbishop of Baltimore; Philander P. Claxton, A. M., Litt. D., United States Commissioner of Education; John H. Finley, LL.D., President of the College of the City of New York; Adelbert Moot, Regent of University of State of New York; Sir James Grant, M. D., K. C. M. G., Ottawa; Dr. M. Uribe Troncoso, Chief, Department of School Hygiene, Mexico, D. F.; Dr. Rupert Blue, Surgeon General, U. S. Public Health and Marine Hospital Service; Dr. H. M. Bracken, Secretary and Executive Officer, Minnesota State Board of Health; Dr. Andrew S. Draper, Commissioner of Education, State of New York; Dr. Theobald Smith, Professor of Comparative Pathology, Harvard Medical School, Boston, Mass.; David Starr Jordan, President, Leland Stanford, Jr., University; Dr. Henry R. Hopkins, Professor Emeritus of Hygiene, University of Buffalo, representing Buffalo Academy of Medicine; Dr. Woods Hutchinson, representing National Education Association; Secretary--General, Dr. Thomas A. Storey, Professor of Hygiene, College of the City of New York, New York City, U. S. A.; Treasurer-General, John H. Lascelles, Vice-President, Marine National Bank, Buffalo, New York, U. S. A.; Executive Committee, Dr. Arthur T. Cabot, Chairman, Fellow, Harvard College (deceased); Dr. Francis E. Fronczak, Commissioner of Health, Buffalo, N. Y.; Dr. Robert W. Lovett, Assistant Professor of Orthopedic Surgery, Harvard Medical School; Henry P. Emerson, Superintendent of Education, Buffalo, N. Y.; Dr. Luther H. Gulick, Director, Department of Child Hygiene, Russell Sage Foundation, New York City; Harold J. Balliett,

City Clerk, Buffalo, N. Y.; Dr. David L. Edsall, Professor of Preventive Medicine, Harvard Medical School, Boston, Mass.; John H. Lascelles, Vice-President, Marine National Bank, Buffalo, New York, U. S. A.; Joseph Lee, Boston, Mass.; Dr. Thomas A. Storey, Secretary.

There is now being arranged a comprehensive program of papers and discussions covering the entire field of school hygiene. There will be scientific exhibits, representing the best that is being done in school hygiene, as well as commercial exhibits of practical and educational value to school people. Nor will the entertainment of the delegates in any way be a minor feature. Plans are being made for a series of social events, including receptions and a grand ball, a pageant in the park, and excursion trips to the great industrial plants of Buffalo, as well as to the wonders of Niagara Falls and the Rapids. Buffalo itself has just taken up a collection of $40,000 for the purpose of covering the expense of the Congress. Delegates will attend from all the leading nations, from every college and university of note in this country, and from various other educational, scientific, medical and hygienic institutions and organizations. The Congress is further open to all persons interested in school hygiene. Membership may be secured on the payment of a five dollar fee. Applications should be sent to Dr. Thomas A. Storey, College of the City of New York, New York City.

---

Preliminary Program of the Sixteenth Annual Meeting of the American Gastro-Enterological Association, Washington, D. C., May 5 and 6, 1913: President's Address, "Remarks on the Role which Functional Disorders Play in the Pathology of the Stomach," J. Kaufmann, New York City; "A Contribution to the Bacteriology of the Duodenum," Arthur F. Chace, New York City; "The Beneficial Effect of Duodenal Alimentation in Cirrhosis of the Liver," Max Einhorn, New York City; "Polyadenoma Gastrica," Jesse S. Myer, St. Louis, Mo.; "Report of a Case of Chronic Stenosing Gastritis," H. W. Soper, St. Louis, Mo.; "Contribution to the Significance of Abdominal Pain," L. Kast, New York City; "Fluoroscopic Examination Versus Clinical Methods in the Diagnosis of Gastric Diseases," John Dudley Dunham, Columbus, O.; "The X-ray Method in Diagnosis of Peptic Ulcer," Franklin W. White and Ariel W. George, Boston, Mass.; "Radiological Studies of the Ileo-cecal Region," A. Holding, New York City; "Gastro-Intestinal Stasis," J. A. Lichty, Pittsburgh, Pa.; "The Frequency of the Transition of Ulcer of the Stomach into Cancer," J. Friedenwald, Baltimore, Md.; "A

Clinical Study of the Relation Between Gastric Ulcer and Gastric Cancer," Frank Smithies, Rochester, Minn.; "Section of the Diaphragm as an Aid in the Exposure of the Thoracic Aorta," Fred Murphy, St. Louis, Mo.; (a) "Esophageal Symptoms in Gastric Disease" (b) "The Employment of Carmin in Gastro-Intestinal Diagnosis," Seymour Basch, New York City; "Lymphoedema: Elephantiasis, Ascites, Obstruction at or near Receptaculum, Non-Filarial," A. L. Benedict, Buffalo, N. Y. All sessions at the New Willard Hotel. Physicians are cordially invited.

---

Historical Medical Exhibition, London, 1913.—Among other historical medical objects of exceptional interest that have been secured for the Historical Medical Exhibition, organized by Mr. Henry S. Wellcome, and which will be opened in London during the meeting of the International Medical Congress in the coming summer, are many relics of Dr. Edward Jenner, the discoverer of vaccination. These include the original lancets and scarifiers he employed during his first experiments, his case and account books, his snuff box, medicine chest and many other interesting articles. A large collection of autograph letters of Jenner's, some of unique interest, have also been loaned, together with the arm chair from his study and in which he died. Other objects connected with the life of Jenner are also to be exhibited, including many valuable portraits of himself and family, painted at different periods, the illuminated addresses presented to him together with the freedoms of the cities of London and Dublin, also medals, and other documents of special interest.

Concerning the history of anaesthesia, many interesting relics are to be exhibited, beginning with the original autograph journal and manuscripts of Henry Hill Hickman, F. R. C. S., the discoverer of the application of the principle of anaesthesia by inhalation for surgical operations, which he proved by actual experiments on animals in 1823. Personal relics of Sir James Simpson, and some of the earliest forms of apparatus for administering chloroform and ether will constitute an exhibit of more than usual interest.

Those who may possess any objects of a similar character connected with the history of medicine and the allied sciences, and who would be willing to loan them, should communicate with the Secretary, 54A, Wigmore street, London, W., England, who will be pleased to forward a complete illustrated catalogue to anyone interested.

An English-Speaking Conference on the Prevention of Infant Mortality will be held in Caxton Hall, Westminster, London, August 4th and 5th. The meetings will be held under the auspices of the (British) National Association for the Prevention of Infant Mortality and the Welfare of Infancy, under the patronage of the King and Queen, and will convene immediately preceding the opening of the International Medical Congress.

Tentative program: The Responsibility of Central and Local Authorities in Infant and Child Hygiene, The Administrative Control of the Milk Supply, The Necessity for Special Education in Infant Hygiene, Medical Problems in Infant Nutrition, Ante-Natal Hygiene.

The American Committee, in charge of the part to be taken by the United States and Canada, will furnish information to those desiring to attend the conference. Dr. Henry L. Coit, Chairman, 277 Mt. Prospect avenue, Newark, N. J.; Dr. Philip Van Ingen, Secretary, 125 East 71st street, New York City.

---

State Society Meeting. The preliminary program was received just too late for our April issue and the May issue will be on the press during the meeting. By June the news items will have been known to the great majority of our readers and the scientific papers will probably have been begun in the State Journal. It is, therefore, from no lack of interest in or loyalty to the State Society that our references to the meeting have been limited to a notice on the cover and a brief allusion in the department of Society Meetings, in our April issue. On the contrary, it seems fairer both to our own subscribers, all of whom we wish to be affiliated with the professional organization of the county, state and nation and most of whom are, and to the State Journal not to duplicate the contents of the latter. The Rochester men are making elaborate preparations for the meeting and we wish them and their guests good weather, a large attendance and an enthusiastic professional spirit such as will justify other cities of the state in further departures from the precedent of holding meetings at the Capital.

---

The Alumni Association of the University of Michigan of Rochester, held a large and enthusiastic dinner-meeting last month, under the presidency of Dr. John R. Williams. Music and two "playettes" enlivened the occasion. Dr. Victor C. Vaughan, Dean of the Medical Department, spoke before the City Club in the afternoon, on the Prevention of Disease, discussing both infections and those due to general developmental factors, as feeble-mindedness. He approved the policy of sterilizing degenerates and of facilitating public hygienic control by division into districts, as contemplated for New York and Michigan.

---

The Medical and Surgical League of Buffalo, at their April meeting, listened to an interesting paper by Dr. Edmund Reimann, entitled, "Cough as a Symptom." Nominations of officers for the ensuing year were made.

---

Buffalo Society of Sanitary and Moral Prophylaxis met April 18 to take action in regard to Senate Bill No. 81, "An Act to amend the domestic relations law in relation to the issuing of marriage licenses." This act is embodied in twenty or thirty lines, and the gist of it is that "No license to marry, referred to in the preceding section shall be issued except after each party to the contract has presented to the license clerk a certificate from a doctor of medicine duly authorized to practice medicine in the State of New York, stating that said doctor of medicine has examined such applicant for a marriage license, and that said applicant is not afflicted with any venereal disease."

The drift of the discussion was to the effect that this proposed law was not perfect, and, in fact, it would be difficult to frame any bill which would fulfill all the desired conditions. But on the other hand the educational value of any such law would be enormous if only to teach the public the necessity of guarding against these diseases and their terrible results in the acquired and inherited forms. Several eminent clergymen also joined in the discussion, and at the end the following resolution was unanimously adopted:

"That it is the sense of this society that we favor Senator Duhamel's Bill No. 81, and urge our representatives in the Senate and Assembly to use their utmost endeavors to have this bill reported favorably, and then further its passage. Also, that steps be taken to acquaint the public with the provisions of this measure in order that it may secure the general approval which it deserves."

It is interesting and gratifying to observe the increasing interest which doctors, clergymen and the public in general are taking in this matter of preventing one of the most terrible scourges to the human race.

Every doctor who knows a legislator should take the first opportunity to preach the gospel of reform. If the bill does not become a law this year, it must pass later. The medical profession and enlightened public sentiment will demand it.

